# Host genetics and viral load in primary HIV-1 infection: clear evidence for gene by sex interactions

**DOI:** 10.1007/s00439-014-1465-x

**Published:** 2014-06-27

**Authors:** Xuelin Li, Matthew A. Price, Dongning He, Anatoli Kamali, Etienne Karita, Shabir Lakhi, Eduard J. Sanders, Omu Anzala, Pauli N. Amornkul, Susan Allen, Eric Hunter, Richard A. Kaslow, Jill Gilmour, Jianming Tang, IAVI Africa HIV Prevention Partnership

**Affiliations:** 1 Department of Medicine, University of Alabama at Birmingham, 1665 University Boulevard, Birmingham, AL 35294 USA; 2 International AIDS Vaccine Initiative, New York City, NY USA; 3 Department of Epidemiology and Biostatistics, UCSF, San Francisco, CA USA; 4 Department of Epidemiology, University of Alabama at Birmingham, Birmingham, AL USA; 5 MRC/UVRI Uganda Virus Research Unit on AIDS, Masaka Site, Masaka, Uganda; 6 Projet San Francisco, Kigali, Rwanda; 7 Zambia-Emory HIV-1 Research Project, Lusaka, Zambia; 8 Centre for Geographic Medicine Research, Kenya Medical Research Institute (KEMRI), Kilifi, Kenya; 9 Centre for Clinical Vaccinology and Tropical Medicine, University of Oxford, Headington, UK; 10 Kenya AIDS Vaccine Initiative (KAVI), Nairobi, Kenya; 11 Department of Pathology and Laboratory Medicine, Emory University, Atlanta, GA USA; 12 Vaccine Research Center, Emory University, Atlanta, GA USA; 13 International AIDS Vaccine Initiative, Human Immunology Laboratory, Chelsea and Westminster Hospital, London, UK; 14 Present Address: Department of Veterans Affairs, Washington, DC, 20420 USA

## Abstract

Research in the past two decades has generated unequivocal evidence that host genetic variations substantially account for the heterogeneous outcomes following human immunodeficiency virus type 1 (HIV-1) infection. In particular, genes encoding human leukocyte antigens (HLA) have various alleles, haplotypes, or specific motifs that can dictate the set-point (a relatively steady state) of plasma viral load (VL), although rapid viral evolution driven by innate and acquired immune responses can obscure the long-term relationships between HLA genotypes and HIV-1-related outcomes. In our analyses of VL data from 521 recent HIV-1 seroconverters enrolled from eastern and southern Africa, HLA-A*03:01 was strongly and persistently associated with low VL in women (frequency = 11.3 %, *P* < 0.0001) but not in men (frequency = 7.7 %, *P* = 0.66). This novel sex by HLA interaction (*P* = 0.003, *q* = 0.090) did not extend to other frequent HLA class I alleles (*n* = 34), although HLA-C*18:01 also showed a weak association with low VL in women only (frequency = 9.3 %, *P* = 0.042, *q* > 0.50). In a reduced multivariable model, age, sex, geography (clinical sites), previously identified HLA factors (HLA-B*18, B*45, B*53, and B*57), and the interaction term for female sex and HLA-A*03:01 collectively explained 17.0 % of the overall variance in geometric mean VL over a 3-year follow-up period (*P* < 0.0001). Multiple sensitivity analyses of longitudinal and cross-sectional VL data yielded consistent results. These findings can serve as a proof of principle that the gap of “missing heritability” in quantitative genetics can be partially bridged by a systematic evaluation of sex-specific associations.

## Introduction

In the era of genome-wide association studies (GWAS) on human traits and diseases, one overwhelming issue is “missing heritability,” as thousands of GWAS (http://www.genome.gov/gwastudies/) have readily identified and confirmed quantitative trait loci (QTLs) based on statistical significance, but these QTLs typically explain little or rather limited phenotypic variance (Brookfield 2013). Proponents of quantitative genetics have called for close attention to study design (Putter et al. 2011), phenotypic robustness (Queitsch et al. 2012), and the effects of rare (including de novo) variants, haplotypes (combinations of variants that are inherited as a single unit), gene by gene interaction (epistasis), gene by environment interaction, as well as epigenetics (Eichler et al. 2010; Gianola et al. 2013; Keller et al. 2012; Lee et al. 2011; Mahachie John et al. 2011). For complex traits with evolving and multifactorial mechanisms, the journey ahead for finding the missing heritability can be long and bumpy.

During the natural course of human immunodeficiency virus type 1 (HIV-1) infection, viremia and time from infection to development of severe immunodeficiency or AIDS are often used as quantitative traits to gauge HIV-1 pathogenesis and/or rates of disease progression. In particular, plasma viral load (VL) set-point during chronic HIV-1 infection offers a relatively steady and widely available outcome measure with both clinical and epidemiological implications (Fideli et al. 2001; Lyles et al. 2000; Mellors et al. 1995; Quinn et al. 2000; Saag et al. 1996). Predictors of set-point VL range from viral characteristics (e.g., subtypes and replicative capacity) (Prentice et al. 2014a; Prince et al. 2012; Yue et al. 2013) to host genotypes (QTLs) that govern innate and adaptive immune responses (Apps et al. 2013; Fellay et al. 2009; Leslie et al. 2010; Prentice and Tang 2012). Depending on the study population and definition of set-point VL (single or multiple measurements), the proportion of VL variance explained by any single host or viral factor is often less than 4 % (Fellay et al. 2007; Prentice et al. 2014a; Yue et al. 2013). The most promising model that incorporates genetic and non-genetic features of epidemiologically linked HIV-1 transmission pairs (source and recipient partners) can account for nearly 37 % of early set-point VL variance (Yue et al. 2013).

Our recent data from a large cohort of HIV-1 seroconverters (SCs) suggest that host and viral factors associated with set-point VL can evolve as the infection progresses (Prentice et al. 2014a), even during the early chronic phase when complications by coinfections and comorbidities are infrequent. The correlates of longitudinal and cross-sectional VL in this cohort include four *HLA*-*B* variants (B*18, B*45, B*53, and B*57) that encode polymorphic cell surface glycoproteins specializing in antigen presentation (Prentice et al. 2014). While these observations are consistent with the well-documented hypothesis that viral epitopes bound to HLA-B molecules can dominate the induction of HIV-1-specific, cytotoxic T-lymphocyte responses (Kiepiela et al. 2004, 2007; Rajapaksa et al. 2012) and further dictate viral evolution or adaptation (Goulder and Walker 2012; Kawashima et al. 2009; Leslie et al. 2004; Moore et al. 2002; Rolland et al. 2010), the VL variance explained by individual *HLA*-*B* variants is also limited (ranging from 0.7 to 1.6 %). Our new objective is to refine the analytical approaches and to identify potential interaction terms between sex and HLA variants.

## Subjects and methods

### Study population

Recent HIV-1 seroconverters (SCs) were enrolled from Kenya, Rwanda, Uganda, and Zambia between 2005 and 2011 (Table 1), under a uniform study protocol sponsored by the International AIDS Vaccine Initiative (IAVI) (Amornkul et al. 2013; Price et al. 2011). The procedures for written informed consent and multidisciplinary research activities were approved by institutional review boards at all clinical research centers and participating institutions.

**Table 1:** Characteristics of HIV-1 seroconverters stratified by sex: demographic features, viral subtypes, outcome measures, and major HLA variants of interest

Characteristics^a^	Men	Women	*P* ^b^
No. of subjects	327	194	NA
Age: mean ± SD (year)	32.5 ± 8.8	29.5 ± 7.3	<0.0001
Age ≥40: no. (%)	59 (18.0)	21 (10.8)	0.027
Enrolment site			<0.0001
Kenya	89 (27.2)	14 (7.2)	<0.0001
Rwanda	50 (15.3)	35 (18.0)	0.412
Uganda	69 (21.1)	58 (29.9)	0.024
Zambia	119 (36.4)	87 (44.9)	0.057
EDIs			
Earliest	3/15/2005	2/4/2005	NA
Latest	10/12/2011	6/29/2011	NA
Length of follow-up (months): median (IQR)	29 (21–31)	29 (20–31)	0.376
Eligible visits per person: median (IQR)	10 (8–11)	9 (7–11)	0.370
HIV-1 subtypes			0.040
Subtype A1	123 (37.6)	52 (26.8)	0.012
Subtype C	118 (36.1)	80 (41.2)	0.242
Others (B, D, recombinants, or unknown)	86 (26.3)	62 (32.0)	0.167
Person-visits with eligible viral load (2–36 months)	3,002	1,732	NA
Person-visits with eligible CD4^+^ T-cell counts	3,000	1,777	NA
First eligible viral load (log_10_)^c^: mean ± SD	4.49 ± 1.03	4.30 ± 1.09	0.048
HLA-A*03: no. (%)	25 (7.7)	22 (11.3)	0.155
Kenya	5 (5.6)	1 (7.1)	–
Rwanda	5 (10.0)	5 (14.3)	–
Uganda	5 (7.3)	9 (15.5)	0.140
Zambia	10 (8.4)	7 (8.1)	–
HLA-B*18: no. (%)	23 (7.0)	11 (5.7)	–
HLA-B*45: no. (%)	58 (17.7)	25 (12.9)	0.144
HLA-B*53: no. (%)	60 (18.4)	41 (21.1)	0.437
HLA-B*57: no. (%)	30 (9.2)	18 (9.3)	–
HLA-C*18: no. (%)	26 (8.0)	18 (9.3)	–

^a^Non-standard abbreviations: *IQR* interquartile range (25th to 75 % percentile), *SD* standard deviation of the mean, *NA* not applicable

^b^The *P* values >0.50 are omitted (−)

^c^First eligible outcome beyond the acute phase of infection (>9 weeks after EDI)

### Follow-up strategies, genotyping, and outcome measures

SCs in this study were identified by frequent (monthly to quarterly) testing of HIV-1 seronegative subjects at high risk of HIV-1 infection through heterosexual and homosexual exposure, with the majority being seronegative partners in HIV-1 discordant couples and/or individuals reporting multiple heterosexual partners or diagnosed with sexually transmitted infections (85 % of the SC cohort). The subjects included for this study were SCs with sufficient longitudinal data, and the visit intervals were expanded from 3 to 24 months (Prentice et al. 2014a) to 2 to 36 months beyond estimated dates of infection (EDI). All study visits considered were before the initiation of antiretroviral therapy under national guidelines (Ngongo et al. 2012). Viral sequencing, molecular HLA genotyping, and quantification of plasma VL followed procedures described in detail elsewhere (Amornkul et al. 2013; Prentice et al. 2014a; Price et al. 2011; Tang et al. 2011). Identification of HLA-B*18 (unfavorable), B*45 (unfavorable), B*53 (unfavorable), and B*57 (favorable) as independent correlates of longitudinal or cross-sectional VL in this heterogeneous cohort (Prentice et al. 2014a) was highly consistent with results concerning Africans and African Americans (Apps et al. 2013; Lazaryan et al. 2011; Leslie et al. 2010; Tang et al. 2010).

### Descriptive statistics

HIV-1-infected men and women were compared for their overall baseline characteristics, including (a) Wilcoxon’s rank-sum test for quantitative variables lacking a normal distribution, (b) *t* test for quantitative variables with a normal distribution, and (c) *χ* ^2^ and Fisher exact tests for categorical variables (Table 1). These and other analytical procedures (summarized below) were done using SAS, version 9.3 (SAS Institute, Cary, NC, USA).

### Central hypothesis and analytical procedures

Our study aimed to test a central hypothesis that gene (HLA class I) by sex (viral microenvironment) interaction can be uncovered by separate analyses of men and women, especially when longitudinal VL measurements (with log_10_-transformation) are evaluated in mixed models. Data analyses began with the screening of potential interaction terms, with a focus on common HLA variants (population frequencies ≥4 %). The timing and magnitude of sex-specific effects on VL were further assessed by local regression (LOESS) curves (longitudinal data) and generalized linear models for geometric mean (cross-sectional) VL. Association signals with false discovery rate (FDR) below 0.20 were entered into a series of sensitivity analyses using subsets of data corresponding to (1) the 3- to 24-month follow-up period with densely distributed visits (Prentice et al. 2014a), (2) stepwise elimination of subjects representing individual countries or geographic regions (e.g., eastern versus southern Africa), and (3) elimination of subjects infected with rare or unknown HIV-1 subtypes. In the final multivariable models, age, sex, duration of infection (DOI, measured quarterly), and previously identified (generalizable) HLA variants (B*18, B*45, B*53, and B*57) (Prentice et al. 2014) were treated as covariates. The performance of individual statistical models was gauged by their overall *R* ^2^ values (corresponding to variance explained by factors in the model), while the impact of individual factors was measured by the regression beta (adjusted mean beta difference, Δ*β*, and standard error, SE). Associations with borderline statistical significance (*P* ≤ 0.050, FDR = 0.20–0.50) were exempt from multivariable models or sensitivity analyses.

### Refinement through evaluation of linkage disequilibrium (LD) profiles and extended haplotypes

Using SAS Genetics (SAS Institute, Cary, NC, USA), HLA genotyping data for eastern and southern African SCs were analyzed separately for LD and extended haplotypes, with additional reference to fully resolved haplotypes in other populations (Cao et al. 2001). Association analyses based on 2- and 3-locus haplotypes were deemed informative if the adjusted effect sizes improved over those attributable to the component alleles.

### Bioinformatics

Several public databases were surveyed for existing evidence of function mechanisms pertinent to HLA/MHC gene expression and immune surveillance. First, HLA-restricted HIV-1 epitopes were retrieved from the HIV Molecular Immunology Database (http://www.hiv.lanl.gov/content/immunology/ctl_search, last accessed on May 18, 2014). Second, MHC SNPs known to tag-specific HLA class I alleles in Africans (de Bakker et al. 2006) were queried in HaploReg (Ward and Kellis 2012) for additional LD information uncovered by The 1000 Genomes Project or functional properties annotated by the ENCODE project (Encode Project Consortium et al. 2012; Rosenbloom et al. 2010). Third, previous associations with immune disorders and/or gene expression QTLs (eQTLs) (Fairfax et al. 2012) were checked in the NCBI Global Cross-database (http://www.ncbi.nlm.nih.gov/) and the SCAN database (http://www.scandb.org/newinterface/index.html, last accessed on May 20, 2014), with close attention to cis- and trans-acting eQTLs (Nicolae et al. 2010).

## Results

### Characteristics of men and women in the study population

A total of 521 subjects had sufficient prospective data (three or more visits) during the 2- to 36-month interval after EDI (Table 1). The overall baseline data differed between 327 men and 194 women in terms of (1) age (*P* < 0.0001), country of origin (*P* < 0.0001), HIV-1 subtype (*P* = 0.040), and first available VL (*P* = 0.048). HLA alleles of interest had similar distribution in men and women (*P* = 0.14–0.97) (Table 1).

### Screening for interaction terms between sex and HLA factors

**Fig. 1:**
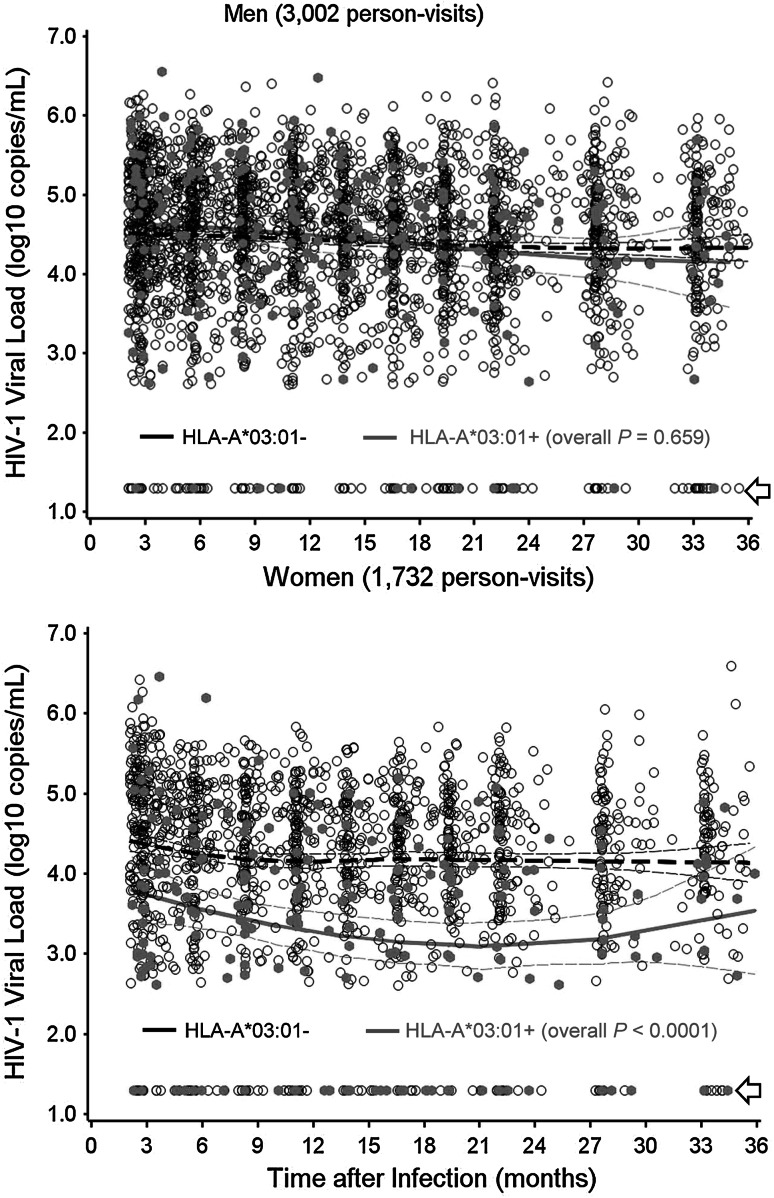
Longitudinal viral loads in HIV-1-infected men and women stratified by HLA-A*03:01. Viral load measurements at various intervals (2 to 36 months after infection) are plotted for HLA-A*03:01-positive and HLA-A*03:01-negative subjects. The *thick and thin lines* correspond to the expected mean value and 95 % confidence intervals for each stratum (see Table 2 for summary statistics based on mixed models). *Arrows* indicate plasma viral load measurements that are <400 RNA copies/ml (routinely transformed to 1.30 log_10_)

When 35 common HLA variants (2- or 4-digit resolution levels, whenever possible) were screened in mixed models with adjustment for demographic factors (age and geography), only HLA-A*03:01 showed a clear interaction with sex (*P* = 0.003, FDR = 0.09). LOESS curves supported this finding, as women with (+) and without (−) HLA-A*03:01 persistently differed in VL over the study intervals (1,732 person-visits, *P* < 0.0001) (Fig. 1). In contrast, HLA-A*03:01+ and A*03:01− men (3,002 person-visits) had highly comparable VL trajectories (*P* = 0.66).

### Multivariable models for longitudinal VL data

For the 2- to 36-month intervals, the interaction term between female sex and HLA-A*03:01 was independent of other known factors pertinent to the study population (Table 2), with an adjusted *P* value of 0.005. On average, VL differed by −0.67 ± 0.24 log_10_ between HLA-A*03:01+ and A*03:01− women after adjusting for other known factors. Analyses of data over the 3–24 months intervals yielded almost identical results (−0.71 ± 0.25 log_10_, *P* = 0.005 for the interaction term) (Table 2).

**Table 2:** Multivariable models for longitudinal viral load (VL) at two overlapping intervals of early HIV-1 infection

Factors in model	Analyses of VL^a^ in the 2–36 months interval				Analyses of VL^a^ in the 3–24 months interval^b^			
	*n*	Δ*β*	SE	Adjusted *P*	*n*	Δ*β*	SE	Adjusted *P*
Age (if >40)^c^	80	−0.06	0.10	0.536	75	−0.10	0.10	0.309
Region (if Zambia)	206	0.36	0.07	<0.0001	199	0.38	0.07	<0.0001
DOI (every 3 months)	521	−0.02	0.01	<0.0001	503	−0.02	0.01	0.007
HLA-B*18	34	0.34	0.14	0.015	34	0.33	0.14	0.023
HLA-B*45	83	0.25	0.09	0.009	82	0.22	0.10	0.027
HLA-B*53	101	0.22	0.09	0.013	93	0.19	0.09	0.047
HLA-B*57	48	−0.48	0.12	<0.0001	46	−0.48	0.12	0.0001
Female sex	194	−0.27	0.07	<0.001	187	−0.29	0.08	<0.001
Female sex × HLA-A*03	22	−0.67	0.24	0.005	22	−0.71	0.25	0.005

^a^Repeated measurements, with log_10_-transformation before analysis. Summary statistics: Δ*β* regression beta (mean deviation, **Δ**, from the reference group), *SE* standard error of the mean Δ*β*

^b^As part of the sensitivity analyses (see text)

^c^For consistency with earlier work, age is retained as a covariate regardless of its statistical significance

### Sensitivity analyses

In separate analyses of subjects representing individual countries or major geographic regions (eastern versus southern Africa), the interaction term for female sex and HLA-A*03:01 was persistently favorable, with adjusted effect sizes (Δ*β*) ranging from −0.30 ± 0.35 log_10_ (*P* = 0.378) to −0.90 ± 0.32 log_10_ (*P* = 0.005) (Table 3), well within the 95 % confidence intervals established by the overall cohort. Data analyses restricted to subjects with different infecting HIV-1 subtypes led to similar observations as well (Table 3).

**Table 3:** Alternative models for evaluating the interaction term between female sex and HLA-A*03

Six alternative models^a^	*n*	Impact on longitudinal VL^b^	
		Δ*β* ± SE	Adjusted *P*
a) Removing Kenyan subjects			
Female sex	180	−0.29 ± 0.08	<0.001
Female sex × HLA-A*03	21	−0.79 ± 0.25	0.002
b) Removing Ugandan subjects			
Female sex	136	−0.31 ± 0.09	<0.001
Female sex × HLA-A*03	13	−0.39 ± 0.28	0.175
c) Removing Zambian subjects^c^			
Female sex	107	−0.22 ± 0.10	0.039
Female sex × HLA-A*03	15	−0.90 ± 0.32	0.005
d) Zambian subjects only^d^			
Female sex	87	−0.34 ± 0.10	0.001
Female sex × HLA-A*03	7	−0.30 ± 0.35	0.378
e) Removing rare HIV-1 subtypes^e^			
Female sex	132	−0.27 ± 0.08	0.001
Female sex × HLA-A*03	13	−0.76 ± 0.28	0.006
f) Removing HIV-1 subtype A1			
Female sex	142	−0.33 ± 0.09	0.0001
Female sex × HLA-A*03	15	−0.53 ± 0.29	0.064

^a^Part of the sensitivity analyses

^b^Repeated measurements in the 2–36 months interval, with log_10_-transformation before analysis. The summary statistics are adjustment for other factors shown in Table 2. *β* regression beta (mean deviation, **Δ,** from the reference group), *SE* standard error of the mean (**Δ**)

^c^The remaining subjects correspond to eastern Africans

^d^Corresponding to southern Africans

^e^Defined as others (not A1 and not C) in Table 1

### Alternative multivariable models for cross-sectional VL data

In a reduced multivariable model, age, sex, geography (clinical sites), previously identified HLA variants (HLA-B*18, B*45, B*53, and B*57), and the interaction term for female sex and HLA-A*03:01 collectively explained 17.0 % of the total variance in the overall geometric mean VL during the 2- to 36-month period (*P* < 0.0001) (Table 4). Statistical adjustments for the number of eligible visits or the length of follow-up for each subject did affect the model (data not shown). The summary statistics remained unchanged in analysis of geometric mean VL during the 3- to 24-month period (Table 4). In this case, the joint model explained 16.2 % of the overall VL variance (Fig. 1).

**Table 4:** Multivariable models for geometric mean viral load (VL) at two overlapping intervals of early HIV-1 infection

Factors in model	Analyses of VL^a^ in the 2–36 months interval					Analyses of VL^a^ in the 3–24 months interval^b^				
	*n*	Δ*β*	SE	Adjusted *P*	*R* ^2^	*n*	Δ*β*	SE	Adjusted *P*	*R* ^2^
Age (if >40)^c^	80	−0.05	0.10	0.585	0.000	75	−0.11	0.10	0.276	0.002
Southern Africa (Zambia)	206	0.35	0.07	<0.0001	0.038	199	0.36	0.08	<0.0001	0.040
HLA-B*18	34	0.35	0.14	0.015	0.010	34	0.35	0.15	0.018	0.010
HLA-B*45	83	0.29	0.10	<0.001	0.014	82	0.27	0.10	0.008	0.012
HLA-B*53	101	0.27	0.09	0.003	0.014	93	0.22	0.10	0.021	0.009
HLA-B*57	48	−0.49	0.12	<0.0001	0.026	46	−0.46	0.13	<0.001	0.022
Female sex	194	−0.28	0.08	<0.001	NA	187	−0.29	0.08	<0.001	NA
Female sex × HLA-A*03	22	−0.72	0.25	0.004	0.014	22	−0.77	0.26	0.003	0.016
Overall	521	NA	NA	<0.0001	0.170	503	NA	NA	<0.0001	0.162

^a^With log_10_-transformation before analysis. Summary statistics: *β* regression beta (mean deviation, **Δ,** from the reference group), *SE* standard error of the mean (**Δ**); *R* ^2^ proportion of VL variance attributable to each factor

^b^As part of the sensitivity analyses

^c^For consistency with earlier work, age is retained as a covariate regardless of its statistical significance

### Other HLA variants of interest

**Fig. 2:**
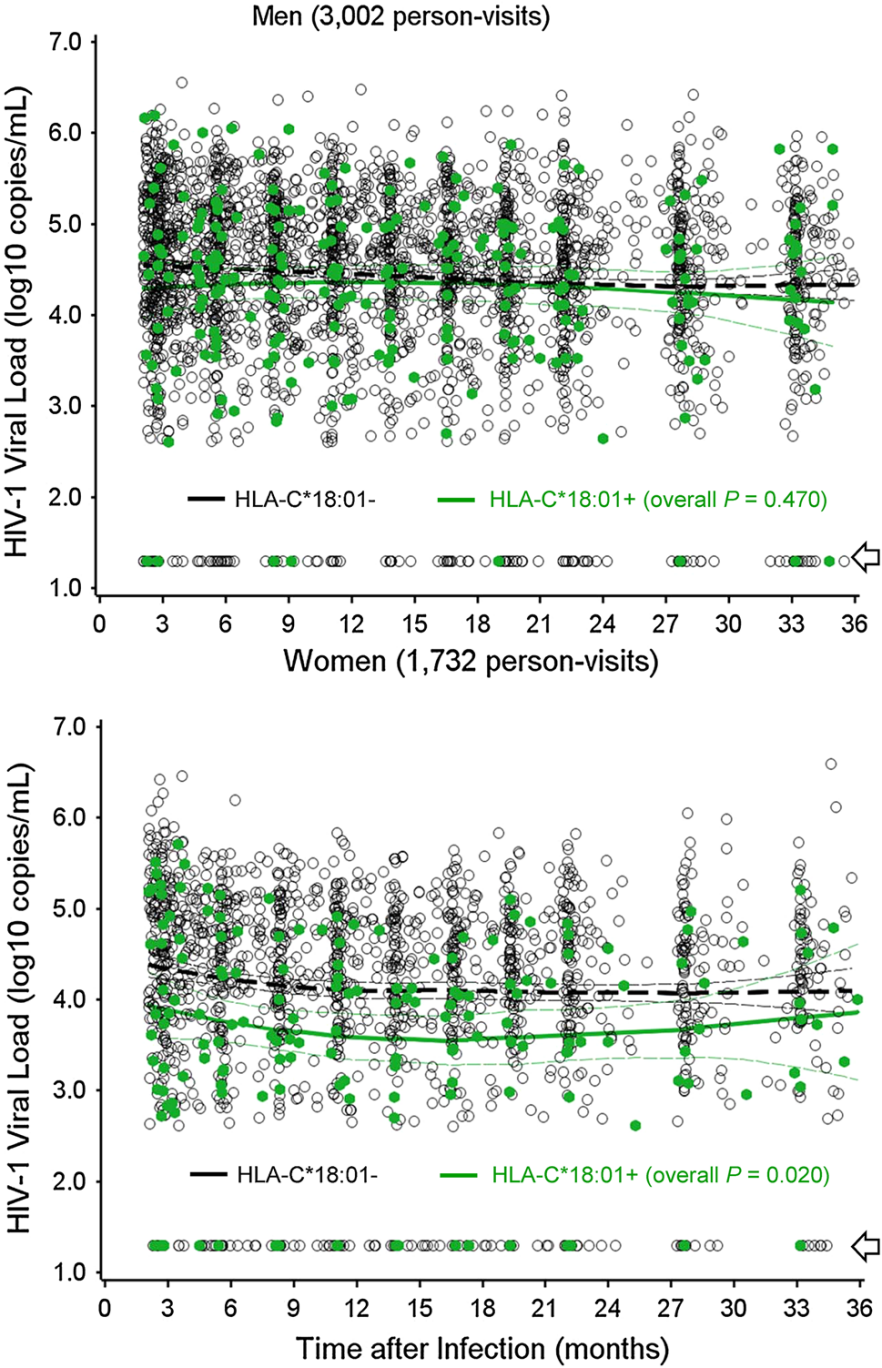
Additional evidence for gene × sex interactions. Prospective viral load measurements are plotted for HLA-C*18:01-positive and HLA-C*18:01-negative subjects. The *thick and thin lines* correspond to the expected mean value and 95 % confidence intervals for each stratum. *Arrows* indicate plasma viral load measurements that are <400 RNA copies/ml (transformed to 1.30 log_10_)

In addition to the main observations on HLA-A*03:01, HLA-C*18:01 showed a trend for favorable interaction with female sex (*P* = 0.042, FDR > 0.50) (Fig. 2). Lack of LD between A*03:01 and C*18:01 (*D*′ = −0.06, *r* ^2^ < 0.001, *P* = 0.90) ruled out the possibility of mutual tagging. Stratification by country did not reveal LD between A*03:01 and C*18:01 either (*P* = 0.34–0.99). Meanwhile, a previously reported, sex-specific effect for HLA-A*74:01 and HIV-1 VL (Koehler et al. 2010) could not be substantiated (*P* > 0.50 for the interaction term), although HLA-A*74:01+ and A*74:01− men did differ slightly in longitudinal VL (Δ*β* = −0.22 ± 0.13 log_10_ for HLA-A*74:01+ men, *P* = 0.131).

### HLA-A*03:01-related haplotypes

In contrast to earlier observations based on five North American populations (Cao et al. 2001), HLA-A*03:01 is not in strong LD with B*07:02-C*07:02 in our study cohort. The only statistically meaningful LD profiles for A*03:01 (*P* ≤ 0.02) involved two haplotypes (B*49:01-C*07:01 and B*58-C*06) present in the eastern African SCs. Neither B*49:01-C*07:01 nor B*58-C*06 had differential impact on VL in women (adjusted *P* = 0.17 and 0.64, respectively). The observed 3-locus haplotypes containing A*03:01 were too diverse (9–11 per geographic region) to allow separate testing.

### HLA-A*03-restricted HIV-1 epitopes

In the context of antigen presentation and CTL responses, multiple studies have identified HLA-A*03-restricted HIV-1 epitopes, especially a conserved epitope (KK9/RK9) in Gag (p17) (Balamurugan et al. 2010; Brumme et al. 2008; Dinges et al. 2010; Goulder et al. 1997; Heath et al. 2011; Peretz et al. 2011; Schneidewind et al. 2009). Other HLA-A*03-restricted CTL epitopes have been mapped to Env/gp120 (TW9 and VE12) (McKinnon et al. 2007; Peretz et al. 2011; Schneidewind et al. 2009), Nef (AK9, GL9, QK10, RK9, and RK10) (Almeida et al. 2011; Balamurugan et al. 2010; Brumme et al. 2008; Peretz et al. 2011; Schneidewind et al. 2009), Pol (AK11, ATK9, DI11, KA9, and SK11) (Balamurugan et al. 2010; Brumme et al. 2008; Chen et al. 2009; Peretz et al. 2011; Turnbull et al. 2009), Rev (TY9) (Yang et al. 2005), and a cryptic antigen (RR9) encoded by an alternative open reading frame (Berger et al. 2010). None of these existing immunologic data have been stratified by sex.

### Further findings from bioinformatics

In populations of African ancestry (e.g., Yoruba), HLA-A*03:01 is tagged by one intergenic SNP (rs2524024), which is in strong LD (*r* ^2^ = 0.81–1.0) with 63 other intergenic SNPs distributed along a 45.1 kb region (5.9–51 kb upstream of *HLA*-*A*). The rs2524024 SNP is also a known eQTL for the integral membrane protein 2A gene (*ITM2A*) at Xq13.3-Xq21.2. When ranked by *P* values, rs2524024 (8.0 × 10^−6^) falls out of the top 20 eQTLs (SNPs) associated with *ITM2A* gene expression in lymphoblastoid cell lines (Nicolae et al. 2010).

## Discussion

By focusing on generalizable findings that are applicable to eastern and southern Africa with multiple circulating HIV-1 subtypes, our analyses yielded clear evidence that female sex can be an important environmental factor to facilitate HLA class I-mediated immune control of HIV-1 infection. Because women typically have lower VL than men after acquiring HIV-1 (Fideli et al. 2001; Prentice and Tang 2012; Tang et al. 2002), our hypothesis about gene by sex interaction may offer some explanation.

In the context of HIV-1 infection, at least two earlier studies have alluded to sex-specific findings with HLA-A*74:01 and HLA-DRB1*11 (Hendel et al. 1999; Koehler et al. 2010). In our analysis, HLA-A*74:01 (a frequent allele) was weakly associated with relatively low VL in men. However, there was no evidence for interaction between HLA-A*74:01 and sex. The second hypothesis about HLA-DRB1*11 being unfavorable in women was derived from a French cohort (Hendel et al. 1999), but analyses of HIV-1-infected Zambians did not replicate that finding (Tang et al. 2010). Unlike earlier studies that did not account for potential false discoveries from random, multiple testing, the interaction term seen here for female sex and HLA-A*03:01 was accompanied by a low FDR (<0.10). A series of sensitivity analyses established that other potential confounders, including age, geography, and viral subtypes, did not obscure or compromise our analytical approaches. Data from the Multicenter AIDS Cohort Study may provide anecdotal evidence to support our key findings, as analyses of viral load and disease progression have never detected differential effects for HLA-A*03 in HIV-1-infected men (Kaslow et al. 1996; Mann et al. 1998).

Statistical significance aside, the threshold for a biologically significant difference in HIV-1 VL is around 0.30 log_10_ after accounting for intra- and inter-assay variability (Modjarrad et al. 2008; Saag et al. 1996). By our estimates, female sex by HLA-A*03:01 interaction was independently associated with ~0.70 log_10_ reduction in VL (Tables 2, 3, 4), which should impact disease progression and vertical or horizontal HIV-1 transmission.

The condition for analyzing gene by sex interactions in our study population was somewhat suboptimal. First, men and women eligible for analyses differed in several non-genetic (and potentially confounding) features (Table 1), which mandates the application of multivariable models and sensitivity analyses. As such, the effect sizes (regression beta and *R* ^2^ values) attributable to specific interaction terms often differed by statistical models and complicated the interpretation process. Second, HLA profile and genetic backgrounds can differ by country and geographic region, suggesting that our emphasis on generalizable findings might have come at the expense of country-specific phenomena. Third, sample size was not equal between men and women, so the statistical power was somewhat compromised in analyses of female-specific associations. As such, the modest trend seen with HLA-C*18:01 in women (Fig. 2) is still worth noting. In the long term, statistical models for gene by sex interactions should continue to improve when homogeneous cohorts with unbiased sex ratios are available for follow-up studies.

HLA alleles that have early influences on HIV-1 viral load tend to impose a strong selection pressure for viral immune escape mutations, as often seen in individuals with HLA-B*57 and related alleles (Bansal et al. 2007; Crawford et al. 2009; Leslie et al. 2004; Novitsky et al. 2010; Wang et al. 2009). In HIV-1-infected African women, the VL trajectory associated with HLA-A*03:01 was relatively steady in the first 3 years of follow-up (Fig. 1). Further evaluation of immune responses and HIV-1 immune escape mutations in HIV-1-infected women with HLA-A*03:01 may provide new insights about durable immune protection against a broad spectrum of HIV-1 subtypes.

Although HLA-A*03:01 itself can play an important role in inducing immune responses to a variety of CTL epitopes, it is also possible that the interaction term seen with A*03:01 actually reflects the function of other variants that operate in a sex-specific fashion. Such genetic variations can be either upstream (telomeric) or downstream (centromeric) from the *HLA*-*A* locus (Vandiedonck and Knight 2009). The LD profiles in our study cohort strongly suggested that genes downstream from the *HLA*-*A* locus, including *HLA*-*C* and *HLA*-*B*, could not explain the A*03:01 effect. Two alternative hypotheses can relate to other genomic regions. First, through strong LD with rs2524024, a *trans*-acting eQTLs for the *ITM2A* gene at Xq13.3-Xq21.2, HLA-A*03:01 can tag various functionally relevant SNPs. The ITM2A product has been shown to regulate CD8 T-cell selection and activation in mice (Kirchner and Bevan 1999). This biological connection can offer a probable mechanism for the observed interaction between HLA-A*03:01 and female sex. The other alternative hypothesis points to a long-range (~4 Mb) LD between A*03 and the C282Y mutation in *HFE*, which is a recessive causal variant for hereditary haemochromatosis (iron overload) in Caucasians (Cardoso and de Sousa 2003; de Bakker et al. 2006; Hanson et al. 2001). However, this is an unlikely explanation as fine mapping using the ImmunoChip array (Illumina, San Diego, CA, USA) has confirmed that haplotype blocks in the MHC region are relatively short in Africans (Prentice et al. 2014b).

Potential interactions between HLA alleles and sex have been reported for several autoimmune disorders and human malignancies (Davis and Dorak 2010; Dorak et al. 1999; Morrison et al. 2010). For *HLA*-*A* variants alone, evidence of sex-specific effect further points to a short sequence motif corresponding to polymorphic amino acid residues 161, 163, and 165 of the HLA-A protein product (Song et al. 2009). This particular sequence motif does not match the ones highlighted in a recent fine-mapping of HLA class I amino acid sequences in HIV-1-infected African Americans (in the absence of stratification by sex) (McLaren et al. 2012). Nonetheless, the *HLA*-*A* locus is often over-shadowed by *HLA*-*B* and *HLA*-*C* in studies of HIV/AIDS (Apps et al. 2013; Fellay et al. 2009; Leslie et al. 2010; Prentice and Tang 2012). If environmental factors indeed dictate how *HLA*-*A* alleles are expressed or regulated, close attention to gene × environment or gene × sex interaction should provide a deeper understanding of “missing heritability” in quantitative genetics.
